# Calcium Positively Mediates Blue Light-Induced Anthocyanin Accumulation in Hypocotyl of Soybean Sprouts

**DOI:** 10.3389/fpls.2021.662091

**Published:** 2021-05-28

**Authors:** Gang Hu, Xiaomeng Yue, Jinxue Song, Guipei Xing, Jun Chen, Haixia Wang, Nana Su, Jin Cui

**Affiliations:** College of Life Sciences, Nanjing Agricultural University, Nanjing, China

**Keywords:** soybean sprouts, calcium, blue light, flavonoid, anthocyanins

## Abstract

Soybean sprouts are a flavorful microgreen that can be eaten all year round and are widely favored in Southeast Asia. In this study, the regulatory mechanism of calcium on anthocyanin biosynthesis in soybean sprouts under blue light was investigated. The results showed that blue light, with a short wavelength, effectively induced anthocyanin accumulation in the hypocotyl of soybean sprout cultivar “Dongnong 690.” Calcium supplementation further enhanced anthocyanin content, which was obviously inhibited by LaCl_3_ and neomycin treatment. Moreover, exogenous calcium changed the metabolism of anthocyanins, and seven anthocyanin compounds were detected. The trend of calcium fluorescence intensity in hypocotyl cells, as well as that of the inositol 1,4,5-trisphosphate and calmodulin content, was consistent with that of anthocyanins content. Specific spatial distribution patterns of calcium antimonate precipitation were observed in the ultrastructure of hypocotyl cells under different conditions. Furthermore, calcium application upregulated the expression of genes related to anthocyanin biosynthesis, and calcium inhibitors suppressed these genes. Finally, transcriptomics was performed to gain global insights into the molecular regulation mechanism of calcium-associated anthocyanin production. Genes from the flavonoid biosynthesis pathway were distinctly enriched among the differentially expressed genes, and weighted gene co-expression network analysis showed that two MYBs were related to the accumulation of anthocyanins. These results indicated that calcium released from apoplast and intracellular stores in specific spatial-temporal features promote blue light-induced anthocyanin accumulation by upregulation of the expression of genes related to anthocyanin synthesis of “Dongnong 690” hypocotyl. The findings deepen the understanding of the calcium regulation mechanism of blue light-induced anthocyanin accumulation in soybean sprouts, which will help growers produce high-quality foods beneficial for human health.

## Introduction

Anthocyanins, derived from the metabolic pathway of phenylpropane, are important nutritional components of fruits and vegetables. The visible color imparted by anthocyanins attracts pollinators to promote plant reproduction (Andersen and Markham, 2005). In addition, anthocyanins are active oxygen scavengers that respond to biotic and abiotic stress, thereby maintaining the normal growth and development of plants (Gould et al., 2002). For humans, anthocyanins are a powerful antioxidant and are widely present in the daily diet to promote health (Bassolino et al., 2013). Studies have shown that consumption of anthocyanins can reduce the risk of cancer, diabetes, and cardiovascular disease (He and Giusti, 2010; Tsuda, 2012).

The mechanism of anthocyanin biosynthesis has been clearly elucidated in many species (Almeida et al., 2007). Many structural genes have been identified to play specific roles in the anthocyanin biosynthetic pathway, namely, early biosynthetic genes chalcone isomerase (CHI), chalcone synthase (CHS), flavanone 3-hydroxylase (F3H), flavonoid 3′-hydroxylase (F3′H), and late biosynthetic genes UDP-glucose: flavonoid 3-O-glucosyltransferase (UFGT), dihydroflavonol 4-reductase (DFR), and anthocyanidin synthase (ANS) (Hichri et al., 2011). Some structural genes are usually regulated by members of the MBW complex, composed of the R2R3-MYB transcription factor, basic helix-lool-helix (bHLH), and WD repeat domain containing proteins (WD40). For example, MdMYB308L physically interacts with MdbHLH33 and enhances its binding to the MdDFR promoter to improve anthocyanin accumulation in apple (An et al., 2020). Few MYBs are involved in the inhibition of anthocyanin biosynthesis. In addition, members of MBW synergistically regulate anthocyanin biosynthesis by interacting with other transcription factors, such as COP1, NAC, and WRKY (Zhou et al., 2015).

Light-emitting diode (LED) light bulbs have recently been used in controlled-environment agriculture to grow vegetables, aiming to regulate the photosynthesis of secondary metabolism (Alrifai et al., 2019). Secondary metabolites in plants are affected by the quality of light. The expression of CRY2/3, SPAs, HY5, and R2R3-MYBs are activated by blue light, leading to anthocyanin accumulation in tea plants (Zheng et al., 2019). Red light promotes the accumulation of proanthocyanidins in strawberry, possibly by inducing the expression of LAR and ANR (Zhang et al., 2018b). Ultraviolet (UV) radiation increases the expression of late biosynthetic genes (VcDFR, VcANS, VcUFGT, and VcMYB) in developing blueberries, causing anthocyanin biosynthesis (Yang et al., 2018).

Calcium is a widespread second messenger that plays a specific role in the development and growth of plants. Many studies have shown that calcium enhances anthocyanin accumulation by the upregulation of genes related to anthocyanin biosynthesis (Zhu et al., 2019). In *Arabidopsis*, changes in endogenous Ca^2+^ levels modulated sucrose-induced sugar uptake, which in turn regulated anthocyanin accumulation (Dong et al., 2012). In a study by Wang et al. (2005), chlorpromazine pretreatment inhibited the activity of CaM, causing a reduction in anthocyanin levels in *Alternanthera bettzickiana* seedlings. A recent study has reported that Ca^2+^/calmodulin (CaM) specifically targeted FvUGT1 at a site partially overlapping with the interdomain linker in grape cell (Peng et al., 2016). Soybean sprouts are a fresh, nutritious microgreen, and can be mass-produced in a short period of time. However, to date, in-depth and comprehensive studies of the calcium-regulated anthocyanin synthesis mechanism in soybean sprouts under blue light are still limited. In this study, we systematically elaborated the regulation mechanism of calcium on anthocyanin synthesis in soybean sprouts under blue light from the perspective of calcium at the tissue and subcellular level, combined with transcriptomics. The findings could contribute to genetic engineering aimed at increasing the anthocyanin content of vegetables.

## Materials and Methods

### Plant Materials and Treatment

Soybean seeds (Glycine max L., cv. “Dongnong 690”) were disinfected with 0.5% NaClO for 30 min and then washed with distilled water. The sterilized seeds were soaked in distilled water for 8 h to facilitate germination. Germinated seeds were then evenly sown in a tray with two layers of gauze to sprout. The sprouts were then exposed to three light qualities (continuous radiation with an intensity of 30 μmol/m^2^/s) with different culture solutions. The different treatments were as follows: (1) D: cultivated with purified water without light; (2) W: cultivated with purified water under white light radiation; (3) B: cultivated with purified water under blue light radiation; (4) B + CaCl_2_: cultivated with 3 mM CaCl_2_ under blue light radiation; (5) B + LaCl_3_: cultivated with 1 mM LaCl_3_ under blue light radiation; and (6) B+ neomycin: cultivated with 1 mM neomycin under blue light radiation. Temperature and relative humidity during cultivation were maintained at 25°C and 80%, respectively.

Uniformly sized sprouts were harvested at 24 and 36 h. Fresh hypocotyls with deeply pigmented parts were randomly sampled for microscopic observation and biochemical measurement. Others were immediately frozen in liquid nitrogen and stored at −80°C for further analysis.

### Anthocyanin Extraction and Measurement

The total anthocyanin content was extracted from the corresponding hypocotyl tissue using an extraction solution (49.9% ddH_2_O, v/v; 50% methanol, v/v; and 0.1% HCl v/v), and measured as described in the previous study (Zhang et al., 2018a).

### Identification of Anthocyanin Profiles

The qualitative analysis of the anthocyanin profile was based on previous study (Zhang et al., 2019). Fresh hypocotyl samples (2 g) were extracted with 6 ml methanol containing 1% (w/v) HCl, and the mixture was sonicated at 20°C for 10 min. The supernatant was collected after centrifugation and filtered with a 0.22-μm nylon membrane for further analysis.

The sample was qualitatively identified using the Xevo G2-XS Q-TOF high resolution mass spectrometer (Waters, Milford, MA, United States). A 2-μL solution was injected into the analytical column (ACQUITY UPLC™ BEH C18, 2.1 × 100 mm, 1.7 μm particle size), and the flow rate was 0.4 ml min^−1^. Solvent A was 0.1% formic acid in water, and solvent B was 0.1% formic acid in acetonitrile. The elution gradient was applied as follows: 0–2 min, 5% B; 2–17 min, 5–95% B; 17–19 min, 95% B; 19–24 min, 95–5% B. Positive ion mode was used with a capillary voltage of 2.5 kV. MS^E^ acquisition modes were adopted for 150-1,E *m*/*z*. Other parameters were set as follows: ramp collision energy, 20–30 eV; source temperature, 120°C; and desolvation gas temperature, 400°C. Masslynx 4.1 was used for data collection and processing (Waters Co., United States). Daidzein (30 μM) was used as an internal standard. The relative content of anthocyanins was expressed as peak area of the sample/standards.

### Calcium Imaging

Calcium in hypocotyl cells was observed using indicator Fluo-3 AM. The hypocotyl tissues with deeply pigmented part were cut into thin slices by hand and loaded with Fluo-3 a.m. in a 20-mm Hank's balanced salt solution (HBSS) buffer for 40 min at 37°C. The stained tissues were washed in a 10-mM 2-[4-(2-hydroxyethyl)-1-piperazinyl] ethanesulfonic acid (HEPES) buffer three times and incubated at 37°C for 10 min in the dark. Calcium signal was assessed using a 488-nm excitation filter in combination with a 525–530 nm emission filter on a confocal laser scanning microscope system (Carl Zeiss, Oberkochen, Germany).

### Cytochemical Localization of Calcium and Ultrastructural Observation

Cytochemical localization of calcium in soybean hypocotyl cells was performed following the method of Jian et al. (1997) with some modifications. Hypocotyl segments (1 mm^3^ cubes) were immersed in fixative solution containing 4% glutaraldehyde and 2% potassium antimonate in 0.1 mol L^−1^ of phosphate buffer (pH 7.6) for 8 h at 4°C. After fixation, the samples were washed three times (10 min each) with 0.1 M potassium phosphate buffer (pH 7.6) containing 2% potassium antimonate, and then fixed in 1% osmium tetroxide for 2 h. The samples were then washed twice in phosphate buffer containing 2% potassium antimonate, and then washed twice with distilled water. Thereafter, the samples were dehydrated in an ethanol series and embedded in EMbed 812 (EMS, New Jersey, United States). The embedded samples were then sectioned with an EM UC7 ultramicrotome (Leica, Germany) at a thickness of 80 nm. Finally, the sections were observed using an H-7650 transmission electron microscope (Hitachi Co., Tokyo, Japan).

### Measurement of IP3 and CaM Content

IP3 content was determined according to the instruction of plant the 1,4,5-trisphosphate kit (GE Healthcare, Chicago, IL, United States). Fresh hypocotyl tissue (0.5 g) was ground into powder in liquid nitrogen and 0.5 ml of 20% perchloric acid was added. Subsequently, the mixture was incubated on ice for 20 min. The precipitated protein was removed by centrifugation (4°C; 2,000 × g; 10 min). Then, the collected supernatant was used to determine the IP3 content. CaM concentration was measured using a plant CaM content assay kit (Kmaels Biotech, Shanghai, China). Fresh hypocotyl tissue (0.5 g) was ground to powder in liquid nitrogen and homogenized in 50 mM Tris-HCl buffer (containing 1 mM egtazic acid [EGTA], 0.5 mM phenylmethylsulfonyl fluoride [PMSF] and 1 mM β-mercaptoethanol). The extract was disintegrated by ultrasonic treatment for 2 min, incubated in a water bath at 95°C for 3 min, and then centrifuged (10,000 × g; 4°C; 20 min). Finally, the supernatant was collected for the analysis of CaM content.

### RNA Extraction, Library Preparation, and RNA-Sequencing

Total RNA was extracted from hypocotyl tissue using the TRIzol RNA plant plus reagent (Tiangen, Beijing, China). RNA quality was assessed using an Agilent 2100 Bioanalyzer (Agilent Technologies, Palo Alto, CA, United States). Samples with an RNA integrity number score > 7.5 were selected for deep sequencing. The RNA-Seq library was constructed using the TruSeqTM RNA sample preparation Kit for Illumina (San Diego, CA, United States) from 24 samples, including hypocotyls harvested at 24 and 36 h in dark, white light, blue light, and blue light plus LaCl_3_ treatment. The library was sequenced on the Illumina HiSeqTM 2,500 sequencing platform by Genedenovo Biotechnology Co., Ltd (Guangzhou, China). Clean reads were obtained by removing reads that contain adapter, with undetermined base, and with low-quality from raw reads. The transcriptome sequencing results are shown in Supplementary Table 3.

### Transcriptome and Quantitative Polymerase Chain Reaction Analysis

The clean reads were aligned to the reference genome of *Glycine max* after the removal of adapters and low-quality sequences. To assess sequence quality, the saturation and gene coverage were analyzed using the RSeQC-2.3.6 software. Fragments per kilobase per million reads (FPKM) was used to determine the expression level of each transcript (Varet et al., 2016). Significantly differentially expressed genes (DEGs) were defined as genes with *P*-adjust < 0.05 and |log2FC |≥ 1. The raw counts of each transcript were compared using DESeq2 software to detect significant DEGs between pairwise comparison. Genetic Output Analysis Tool (GOAT) and R package were used to perform Gene Ontology (GO) enrichment analysis and Kyoto Encyclopedia of Genes and Genomes (KEGG) enrichment analysis (*P*-adjust < 0.05 after Benjamini and Hochberg correction).

Quantitative real-time PCR (qRT-PCR) was carried out on a Mastercycler ep realplex Real-time PCR System (Eppendorf, Hamburg, Germany) using Bestar SYBR Green qPCR Mastermix (DBI, Bioscience Inc., Germany). Reactions were performed at 95°C for 2 min, followed by 40 cycles of 95°C for 10 s, 60°C for 30 s, and 72°C for 30 s. ELF1B was used as the housekeeping gene (Jian et al., 2008). Specific primers were designed using Primer 5 (Supplementary Table 1). Relative gene expression levels were calculated using the 2^−Δ*ΔCT*^ method (Vandesompele et al., 2002).

### Weighted Gene Co-expression Network Analysis

Weighted gene co-expression network analysis (WGCNA) was performed using an R package (Zhang and Horvath, 2005). Module detection and network construction were performed using an unsigned type of topological overlap matrix, a minimal module size of 50, a power β of 10, and a branch merge cut height of 0.7. The module eigengene value was used to evaluate the correlation between the modules and the anthocyanin content of 24 samples. The most significant module was “MM. green” based on 22 genes (Supplementary Table 2) with a WGCNA edge weight > 0.80, which was given using Cytoscape 3.3.0.

### Statistical Analysis

At least three independent experiments were performed for each treatment. Data were expressed as mean ± standard error (SE) after one-way analysis of variance (ANOVA) using the SPSS 17.0 program (SPSS Inc. Chicago, IL, United States). The data were statistically analyzed using the multiple range test of Duncan (*P* < 0.05).

## Results

### Anthocyanin Accumulation in Hypocotyl of Soybean Sprouts

**Figure 1:**
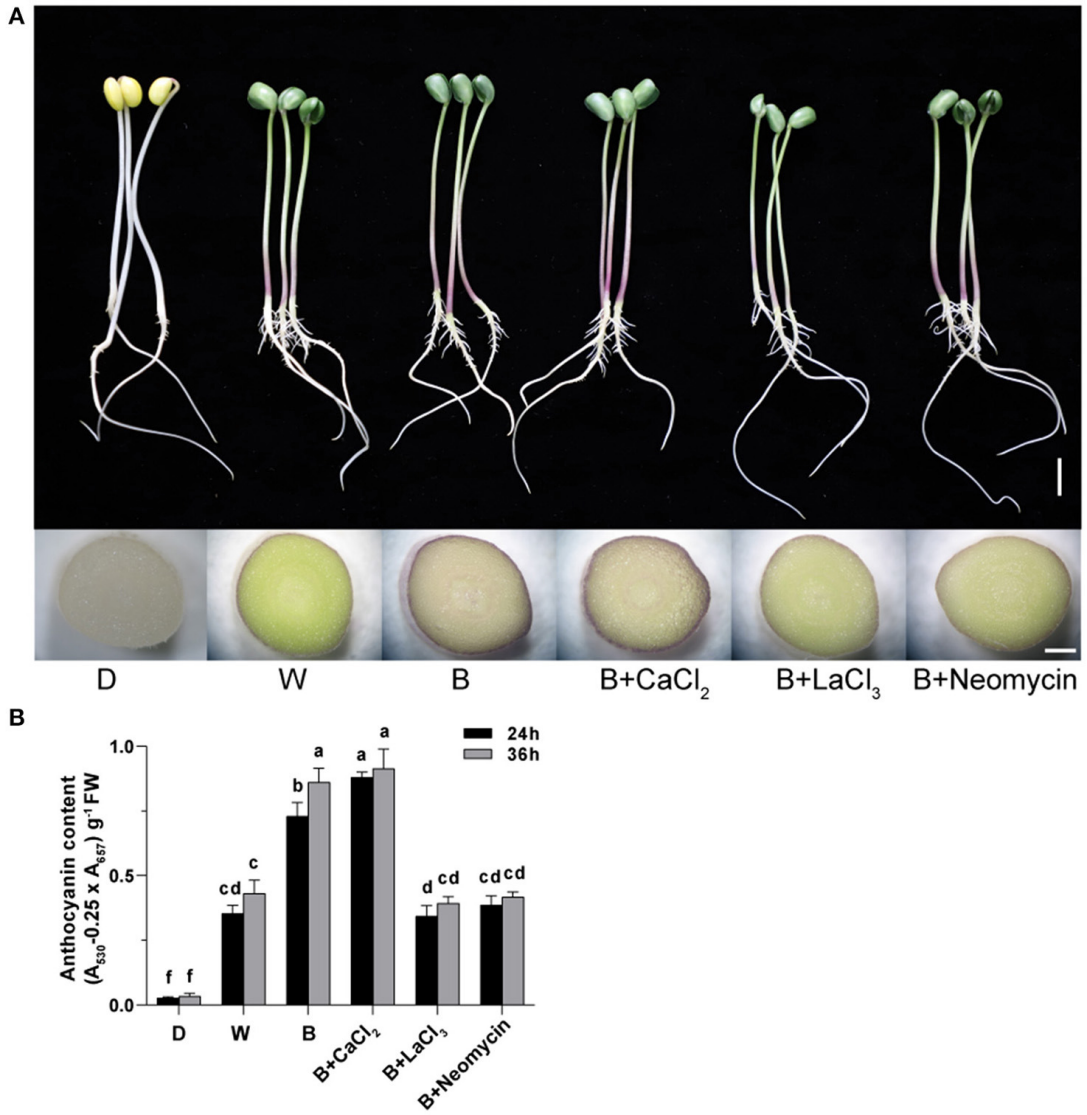
Effects of light, CaCl_2_, and Ca^2+^ inhibitors on phenotype of “Dongnong 690” sprouts and total anthocyanin content in hypocotyl in different treatments. **(A)** Phenotype of “Dongnong 690” sprouts (up panel, bar = 1 cm) and cross section of hypocotyl (down panel, bar = 0.1 cm) under different conditions, 24 h after sowing. **(B)** The total content anthocyanin in hypocotyls, 24 and 36 h after sowing. Values are the mean ± SE of triplicate (*n* = 3). The different letters represent significant differences among various treatments (*p* < 0.05). The different treatments were as follows: (1) D: cultivated with purified water without light; (2) W: cultivated with purified water under white light radiation; (3) B: cultivated with purified water under blue light radiation; (4) B + CaCl_2_: cultivated with 3 mM CaCl_2_ under blue light radiation; (5) B + LaCl_3_: cultivated with 1 mM LaCl_3_ under blue light radiation; and (6) B + neomycin: cultivated with 1 mM neomycin under blue light radiation.

The phenotype of soybean sprouts and total anthocyanin content (TAC) of hypocotyl under different conditions were analyzed (Figure 1). Compared with the D treatment, the W and B treatments caused the hypocotyl to appear purple-red, which was obviously induced to accumulate anthocyanins. Whole areas of the hypocotyl profile in B were deeply colored compared with those in W (Figure 1A). The TAC of the hypocotyl in the D, W, and B treatments were obtained (Figure 1B). The TAC began to be produce rapidly under light irradiation after 24 h. The TAC in B was two-fold higher than that in the W treatment at 24 h. With continuous light exposure of up to 36 h, the TAC in B was further enhanced, but it did not significantly fluctuate in W (Figure 1B). These results indicate that blue light is more effective than white light in inducing the accumulation of anthocyanins in “Dongnong 690” hypocotyls. Furthermore, exogenous calcium significantly increased TAC by 20.5% compared with that of only B radiation at 24 h, but no significant improvement was observed at 36 h. By contrast, the application of LaCl_3_ and neomycin obviously weakened the positive effects of calcium on TAC throughout the experiment. Meanwhile, compared with only B treatment, calcium inhibitors notably prevented the accumulation of anthocyanins (Figure 1B).

### Analysis of Anthocyanin Profile

The composition of anthocyanins from hypocotyl was analyzed using a high resolution mass spectrometer (Table 1). CaCl_2_ treatment promoted the production of a new monomer, pelargonidin 3-O-(6″-succinyl-glucoside), and increased the content of some monomers. For instance, the content of pelargonidin 3-O-galactoside and cyanidin 3-O-(6″-acetyl-galactoside) in the B+ CaCl_2_ treatment was 2.9- and 7.7-fold, respectively, higher than that in the only B treatment. The most abundant compound was pelargonidin 3-O-galactoside in B+ CaCl_2_, with a proportion of 32.4%. In addition to reducing the TAC, LaCl_3_ and neomycin lessened the composition of anthocyanins, with only three components. Date showed that dark conditions did not stimulate anthocyanin accumulation. Under W irradiation, two anthocyanin individuals were detected, of which malvidin 3,5-O-diglucoside only responded to white light. Significant changes based on the anthocyanin composition were observed in B. A total of six anthocyanin individuals were obtained, such as cyanidin, pelargonidin, malvidin, and petunidin derivatives. Among them, pelargonidin-based anthocyanins were predominant, followed by cyanidin-based anthocyanins. The result shown in Table 1 is consistent with the total anthocyanin content in different treatments, as shown in Figure 1B.

**Table 1:** Anthocyanin profile in hypocotyl of “Dongnong 690” sprouts grown in different treatments.

**No**	**Observed m/z**	**Observed RT (min)**	**Compounds**	**Relative abundance**					
				**D**	**W**	**B**	**B+CaCl_**2**_**	**B+LaCl_**3**_**	**B+Neomycin**
1	641.1734	3.02	Petunidin 3,5-O-diglucoside	ND	ND	0.0429 ± 0.0023^b^	0.0948 ± 0.0061^a^	0.0340 ± 0.0029^c^	0.0422 ± 0.0060^b^
2	565.1544	5.34	Pelargonidin 3-O-sambubioside	ND	ND	0.1130 ± 0.0018^b^	0.1380 ± 0.0016^a^	ND	ND
3	565.1544	5.10	Pelargonidin 3-O-galactoside	ND	ND	0.3335 ± 0.0035^b^	0.9689 ± 0.0025^a^	ND	ND
4	533.1284	10.23	Pelargonidin 3-O-(6″-succinyl-glucoside)	ND	ND	ND	0.0630 ± 0.0046	ND	ND
5	271.0602	5.65	Pelargonidin	ND	0.3571 ± 0.0035^d^	0.7797 ± 0.0015^a^	0.6788 ± 0.0152^b^	0.6077 ± 0.0044^c^	0.6193 ± 0.0064^c^
6	493.1323	5.33	Malvidin 3-O-galactoside	ND	ND	0.1271 ± 0.0015^a^	0.1434 ± 0.0021^a^	ND	ND
7	493.1323	3.75	Malvidin 3,5-O-diglucoside	ND	0.2491 ± 0.0034	ND	ND	ND	ND
8	491.1159	11.07	Cyanidin 3-O-(6″-acetyl-galactoside)	ND	ND	0.0411 ± 0.0006^b^	0.3154 ± 0.0038^a^	0.0318 ± 0.0018^c^	0.0288 ± 0.0026^c^
			**Total**		0.6062 ± 0.0015^c^	1.3515 ± 0.0034^b^	2.2126 ± 0.0041^a^	0.6055 ± 0.0020^c^	0.6059 ± 0.0028^c^

*Each value is the mean ± SE (n = 3). Values followed by different letters within the same row are significantly different at P < 0.05 according to the multiple test of Duncan. “ND” means not detected. The different treatments were as follows: (1) D: cultivated with purified water without light; (2) W: cultivated with purified water under white light radiation; (3) B: cultivated with purified water under blue light radiation; (4) B + CaCl_2_: cultivated with 3 mM CaCl_2_ under blue light radiation; (5) B + LaCl_3_: cultivate with 1 mM LaCl_3_ under blue light radiation; and (6) B + neomycin: cultivated with 1 mM neomycin under blue light radiation. 24 h after sowing*.

### Intensity of Calcium in Hypocotyl Profile

**Figure 2:**
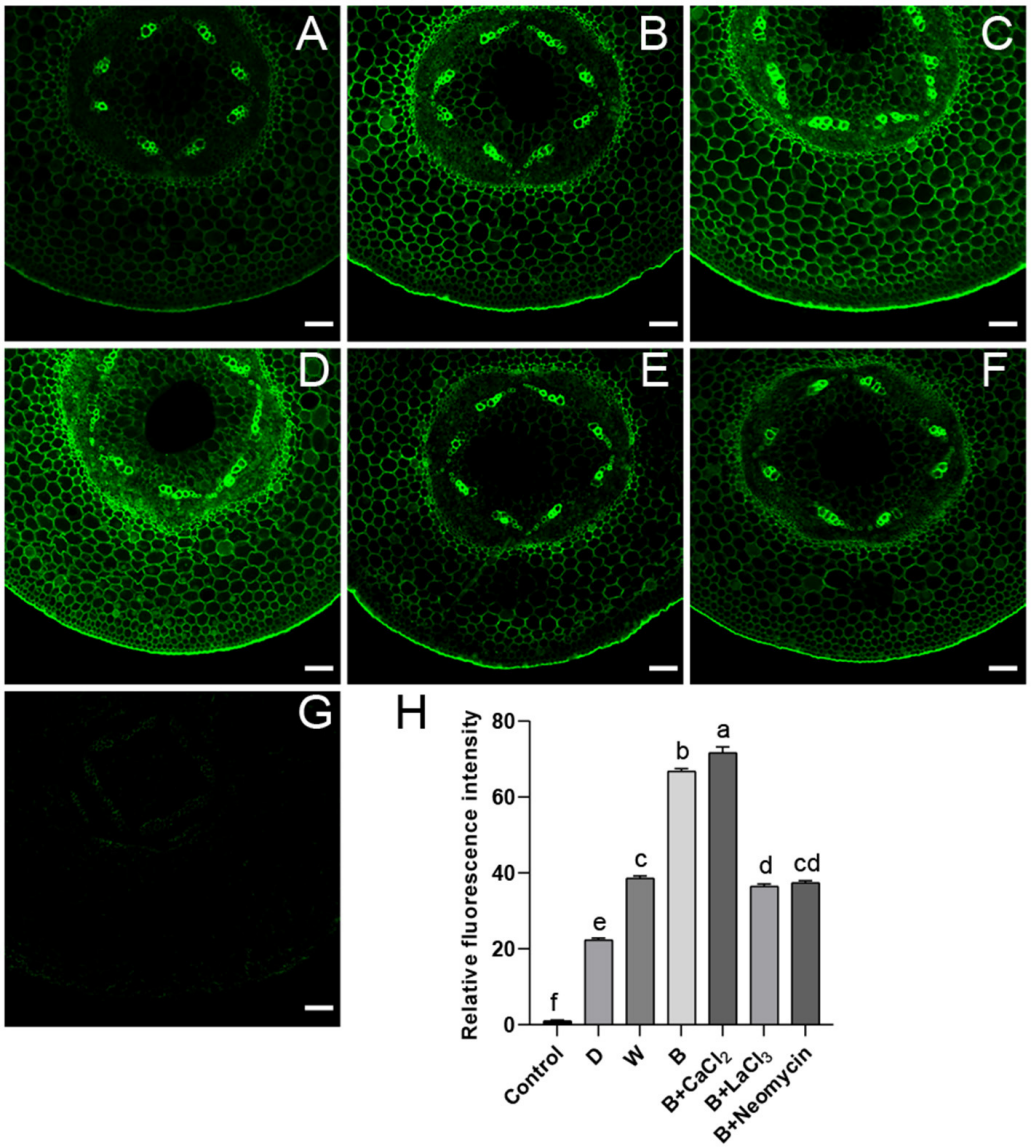
Green fluorescent image of calcium in “Dongnong 690” hypocotyl cells at 24 h under different conditions. **(A–F)** stand for treat with dark, white light, blue light, blue light + 3mM CaCl_2_, blue light + 1mM LaCl_3_, and blue light + 1mM neomycin, respectively. **(G)** Hypocotyl was treated with distilled water without addition of probe. **(H)** Relative fluorescence intensity of calcium in different treatments. bar = 100 μm. Values are the mean ± SE of triplicate (*n* = 3). The different letters represent significant differences among various treatments (*p* < 0.05).

The correlation between calcium and anthocyanin accumulation was investigated using a dye-based calcium indicator (Fluo-3 AM). Hypocotyl not incubated with Fluo-3 AM showed faint spontaneous fluorescence as a control (Figures 2G,H). After CaCl_2_ supplementation in B, the brightest green fluorescent signal was observed, mainly distributed in the stele and endothelial cells of hypocotyl (Figures 2D,H). On the contrary, treated with LaCl_3_ or neomycin, the fluorescence brightness of the hypocotyl section was obviously weakened, showing a distinct inhibitory effect (Figures 2E,F,H). Compared with D, the cross section of hypocotyl in the W and B treatments showed a strong fluorescent signal (Figures 2A–C,H). The relative fluorescence intensity of the hypocotyl profile in B was higher than that in W (Figures 2B,C,H).

### Distribution of Calcium in Hypocotyl Cells

**Figure 3:**
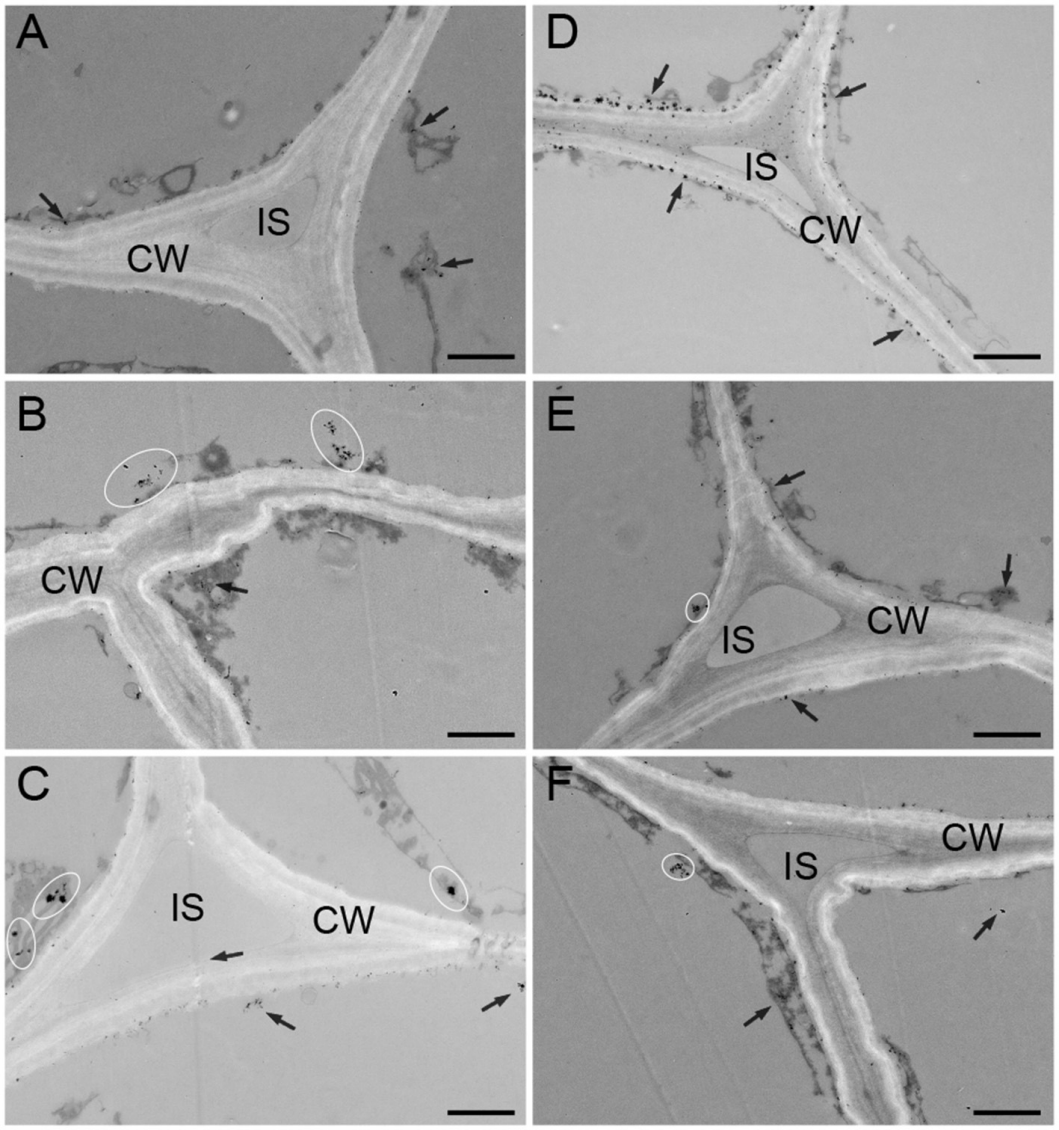
The localization of calcium antimony precipitates in “Dongnong 690” hypocotyl cells at 24 h under different conditions. **(A–F)** stand for treat with dark, white light, blue light, blue light + 3 mM CaCl_2_, blue light + 1 mM LaCl_3_, and blue light + 1 mM neomycin, respectively. bar = 1 μm. Arrows and ovals indicate precipitates of calcium. CW: cell wall. IS: intercellular space.

The distribution of calcium in the subcellular structure of hypocotyl was explored. Calcium antimonate precipitate is an electron-dense particle used to observe calcium localization. Calcium precipitates were distributed in the cytoplasm and near the cell membrane as a small number of tiny irregular particles in D (Figure 3A). In the W treatment, denser sphere-like particles were scattered in the cytoplasm (Figure 3B). The obvious change was that many larger intensive precipitates in agglomerate form were observed in the cytoplasm in B (Figure 3C). After CaCl_2_ supplementation in B, a large amount of sludge deposits appeared around the cell membrane, and a spot of particles was found in the cell wall (Figure 3D). Compared with the B + CaCl_2_ treatment, the black precipitation in the cytoplasm was obviously reduced by the LaCl_3_ and neomycin treatments (Figures 3E,F).

### Determination of IP3 and CaM Content

**Figure 4:**
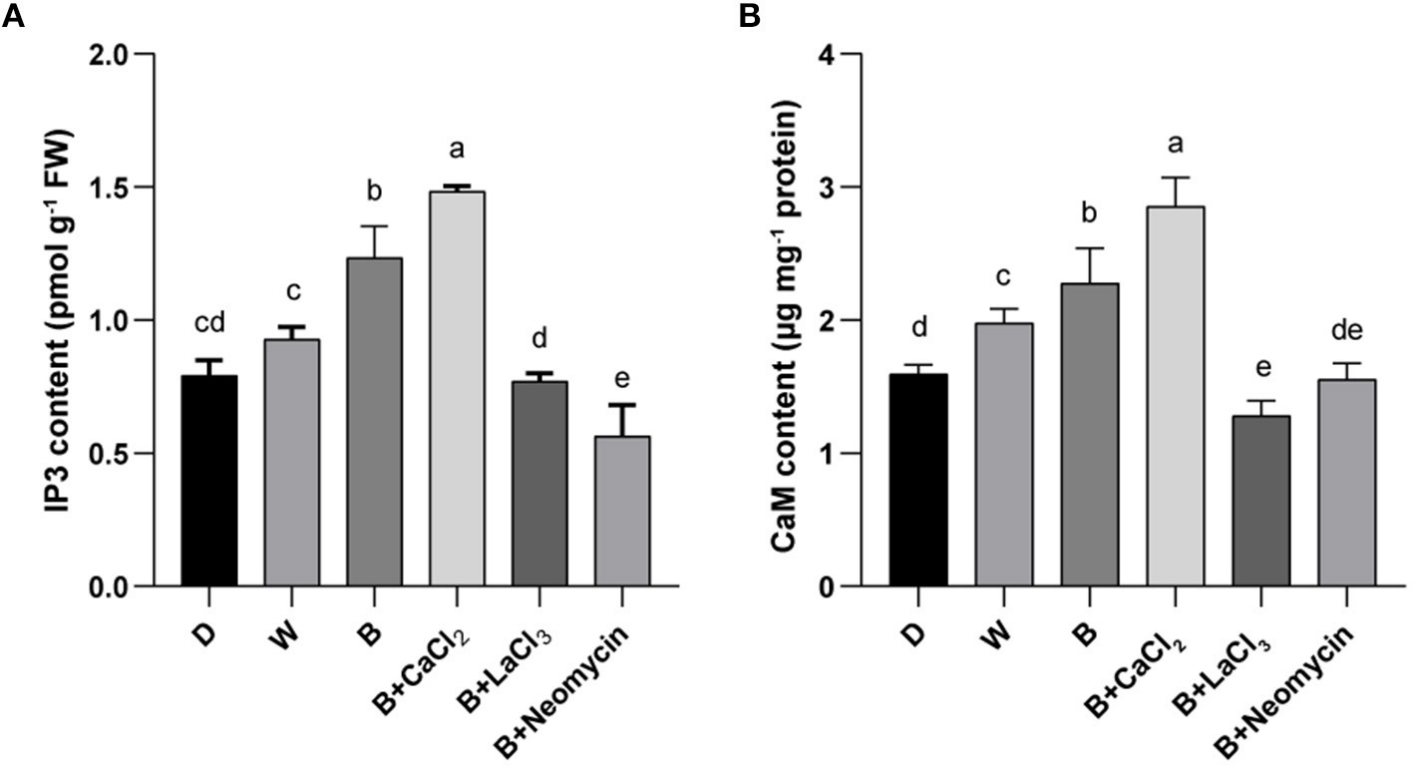
IP3 content **(A)** and CaM content **(B)** in “Dongnong 690” hypocotyls at 24 h under different conditions. Values are the mean ± SE of triplicate (*n* = 3). The different letters represent significant differences among various treatments (*p* < 0.05). The different treatments were as follows: (1) D: cultivated with purified water without light; (2) W: cultivated with purified water under white light radiation; (3) B: cultivated with purified water under blue light radiation; (4) B + CaCl_2_: cultivated with 3 mM CaCl_2_ under blue light radiation; (5) B + LaCl_3_: cultivated with 1 mM LaCl_3_ under blue light radiation; and (6) B + neomycin: cultivated with 1 mM neomycin under blue light radiation.

To determine the role of IP3 and CaM in the calcium signaling pathway for anthocyanin synthesis, their contents were measured. Compared with the blue radiation treatment, exogenous calcium supplementation further increased the IP3 content, which was the highest level among all treatments. On the contrary, LaCl_3_ reduced the IP3 content in the B treatment, reaching a level comparable with that in the D treatment. Neomycin application reinforced this inhibitory effect (Figure 4A). The content of IP3 in hypocotyl in B was higher than that in the D and W treatments (Figure 4A). Similarly, CaCl_2_ application further elevated the CaM content compared with the blue radiation treatment, reaching the highest level. Conversely, treatment with LaCl_3_ resulted in the lowest CaM content, followed by neomycin treatment (Figure 4B). In addition, the CaM content in hypocotyl showed a gradient trend among three light qualities (Figure 4B). Compared with the D treatment, the CaM content in the W and B treatments increased by 24.5 and 43.4%, respectively.

### Transcript Levels of Anthocyanin Biosynthetic-Related Genes

**Figure 5:**
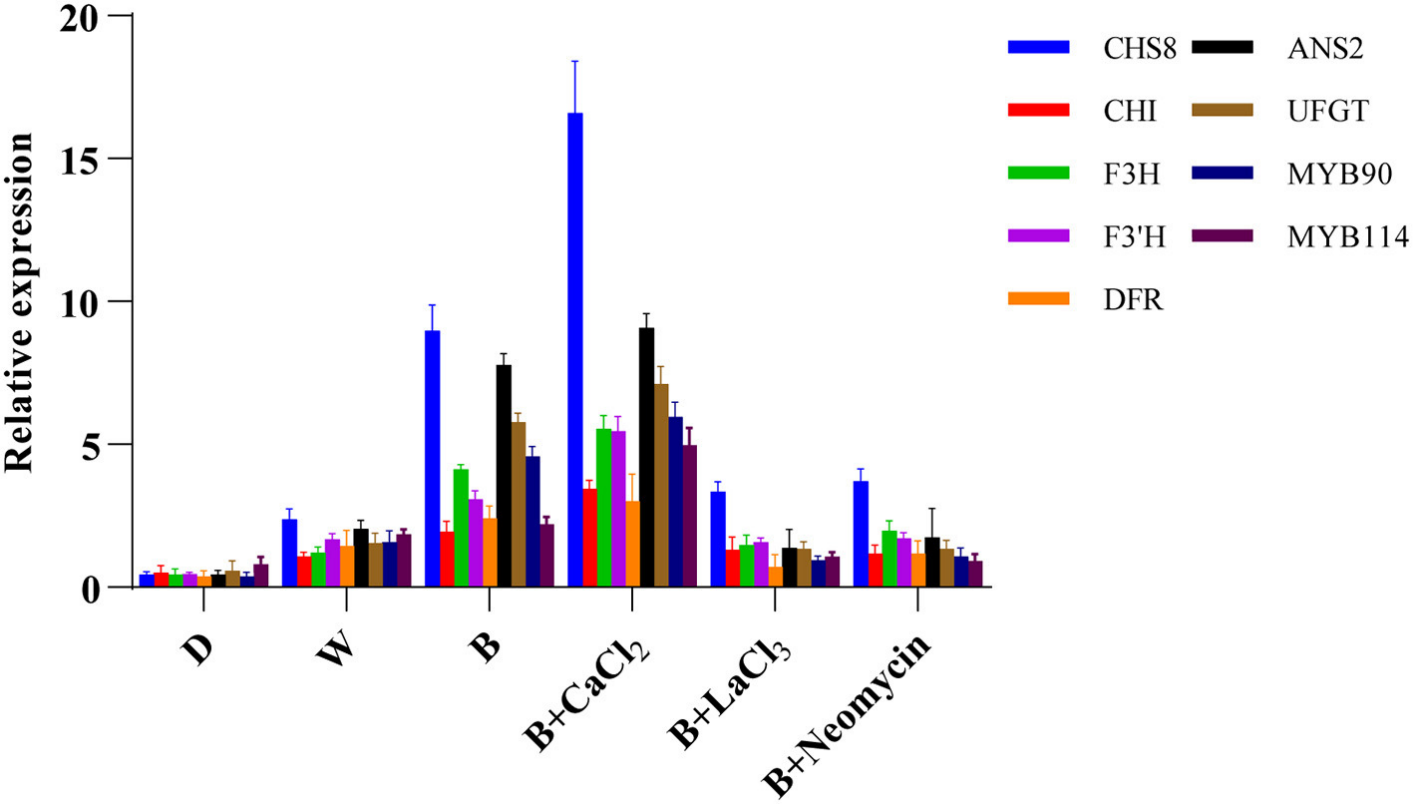
Expression level of genes related to anthocyanin biosynthesis at 24 h in “Dongnong 690” hypocotyls under different conditions. Values are the mean ± SE of triplicate (*n* = 3). The different treatments were as follows: (1) D: cultivated with purified water without light; (2) W: cultivated with purified water under white light radiation; (3) B: cultivated with purified water under blue light radiation; (4) B + CaCl_2_: cultivated with 3 mM CaCl_2_ under blue light radiation; (5) B + LaCl_3_: cultivated with 1 mM LaCl_3_ under blue light radiation; and (6) B + neomycin: cultivated with 1 mM neomycin under blue light radiation.

The expression levels of genes involved in anthocyanin biosynthesis in hypocotyl were investigated using qRT-PCR (Figure 5). Compared with the blue radiation treatment, the transcription level of early biosynthetic genes and late biosynthetic genes were enhanced after CaCl_2_ supplementation, especially those of GmCHS8, GmANS2, and GmUFGT. However, both the LaCl_3_ and neomycin treatments completely reversed this trend. Compared with D, the expression levels of structural genes related to anthocyanin synthesis were upregulated in the W treatment. The B treatment further significantly improved the expression level of these key genes.

### Gene Expression Profiles and Differentially Expressed Genes and Weighted Gene Co-expression Network Analyses

**Figure 6:**
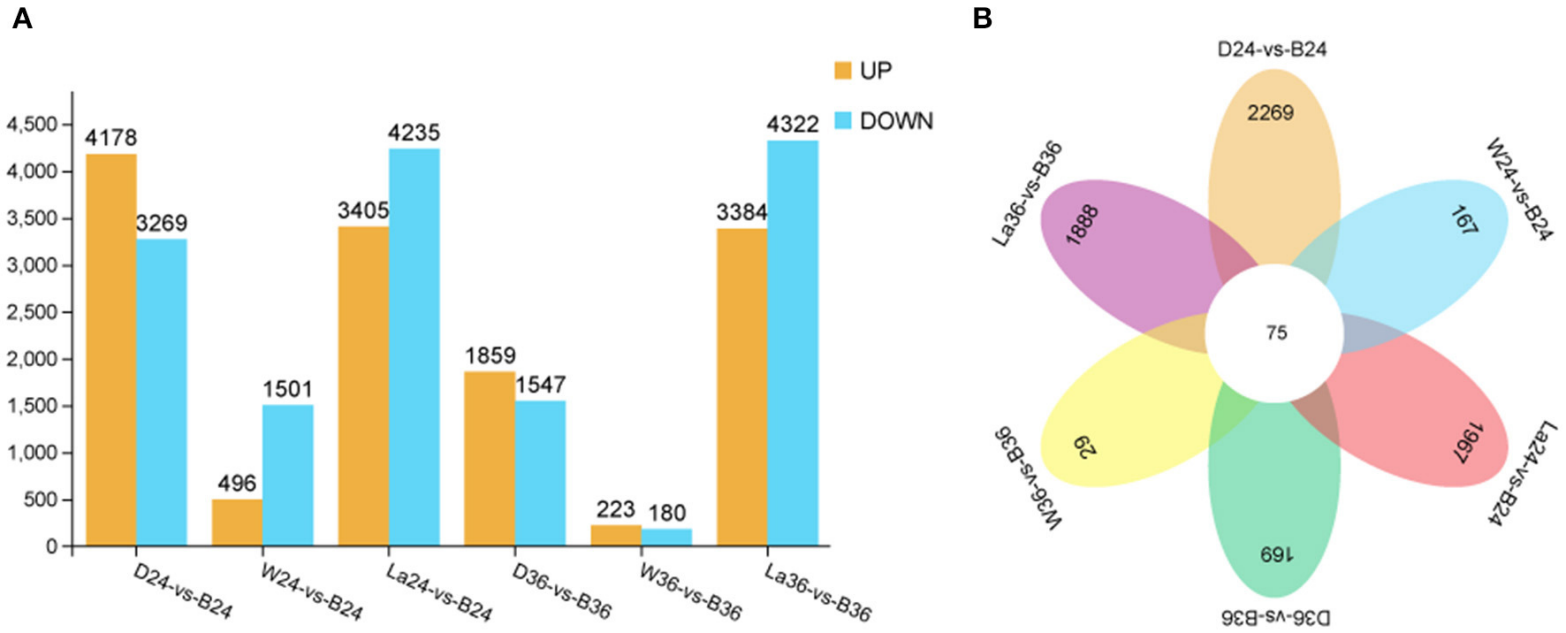
Differentially expressed genes identified by RNA-seq analysis in hypocotyl of sprouts. **(A)** Number of DEGs between D vs B, W vs B, and La vs B groups at 24 and 36 h after illumination. **(B)** Venn diagram representation of total genes from D, W, B, and La groups at 24 and 36 h after illumination. D: dark. W: white light. B: blue light. La: blue light + 1 mM LaCl_3_, 24 or 36h.

To gain global insight into the molecular mechanism of calcium on anthocyanin metabolism, sprouts of hypocotyl samples in the dark, white light, blue light, and blue light + 1 mM LaCl_3_ groups were analyzed at 24 and 36 h by RNA-seq. A total of 28,599 non-redundant DEGs between and among different treatment groups were identified (Figure 6). The total number of DEGs at 24 h was greater than that at 36 h. The results indicate that the early period may be a critical stage that affects the coloration of hypocotyl. Besides, compared with the other pairwise comparison groups, groups La24 vs. B24 and La36 vs. B36 had more DEGs. The expression of 10 selected DEGs from the RNA-Seq data was further verified by qRT-PCR. Linear regression analysis showed that RNA-Seq and qRT-PCR results for these genes were highly correlated (*r* = 0.888–1) (Supplementary Figure 2). These results indicated that the transcriptomic profiling data accurately corresponded to the treatment responses of soybean sprouts hypocotyl.

**Figure 7:**
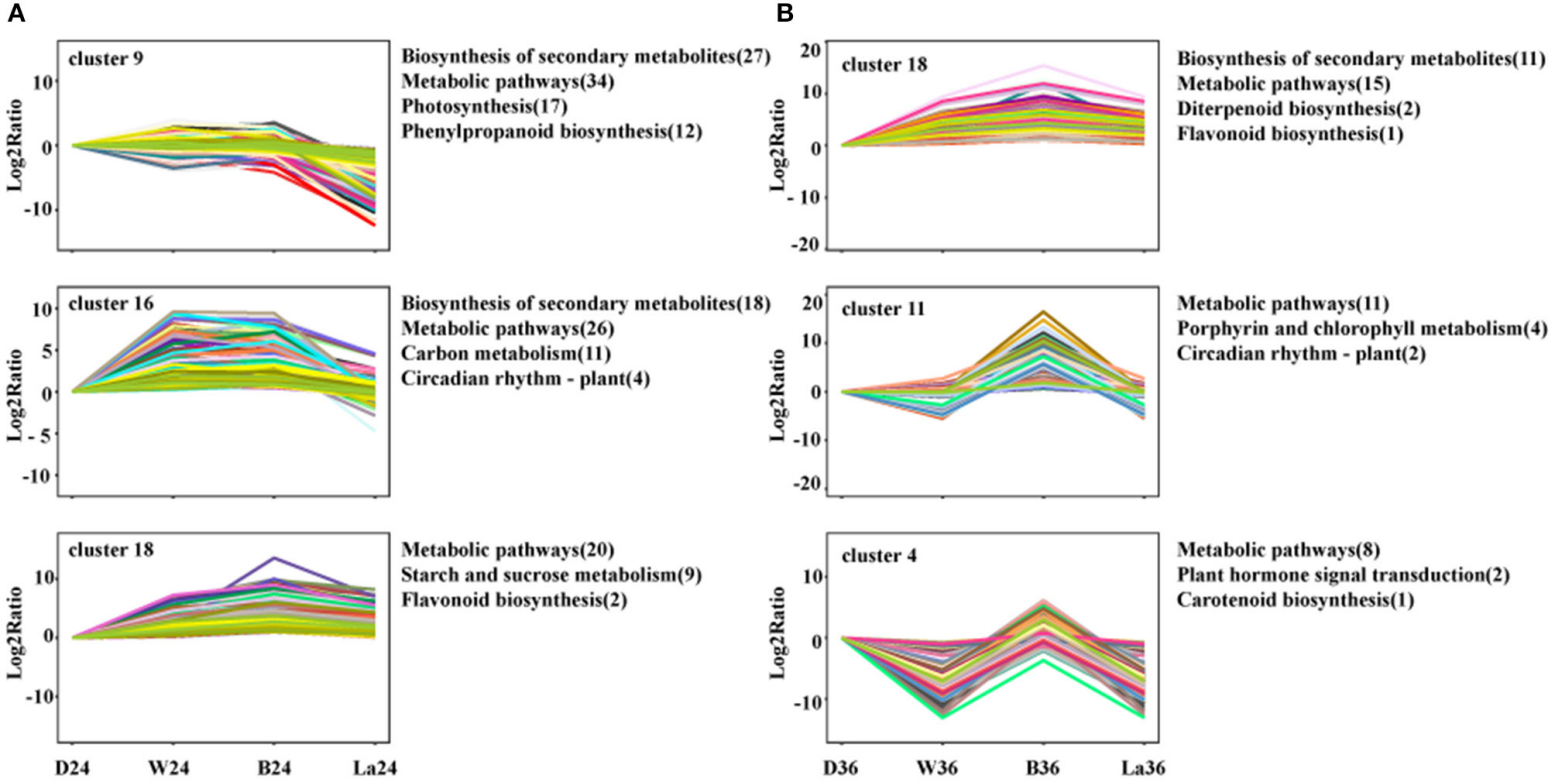
Cluster analysis of DEGs with significant expression profile changes and KEGG pathway enrichment analysis at 24h **(A)** and 36h **(B)**. All the DEGs were subjected to complete-linkage hierarchical clustering using a Euclidean distance metric and divided into 20 clusters at two time points. Enriched KEGG pathways are listed to the right of each cluster. D: dark. W: white light. B: blue light. La: blue light + 1 mM LaCl_3_, 24 or 36 h.

To further understand the relevant biological processes, the transcripts were divided into 20 profiles at each time point (Supplementary Figure 1), representing different expression patterns. Then, KEGG pathway enrichment analysis was performed to comprehensively observe the biological pathways enriched in clusters with similar expression trends. The transcripts in samples treated with LaCl_3_ in clusters enriched in the biosynthesis of secondary metabolites, metabolic pathways, and flavonoid biosynthesis were all downregulated compared with those in the B treatment (Figure 7). Furthermore, flavonoid biosynthesis was enriched in Cluster 18, included in both 24 and 36 h groups, and contained genes with similar expression patterns but with different relative Log2 ratio in the same treatment (Figure 7).

**Figure 8:**
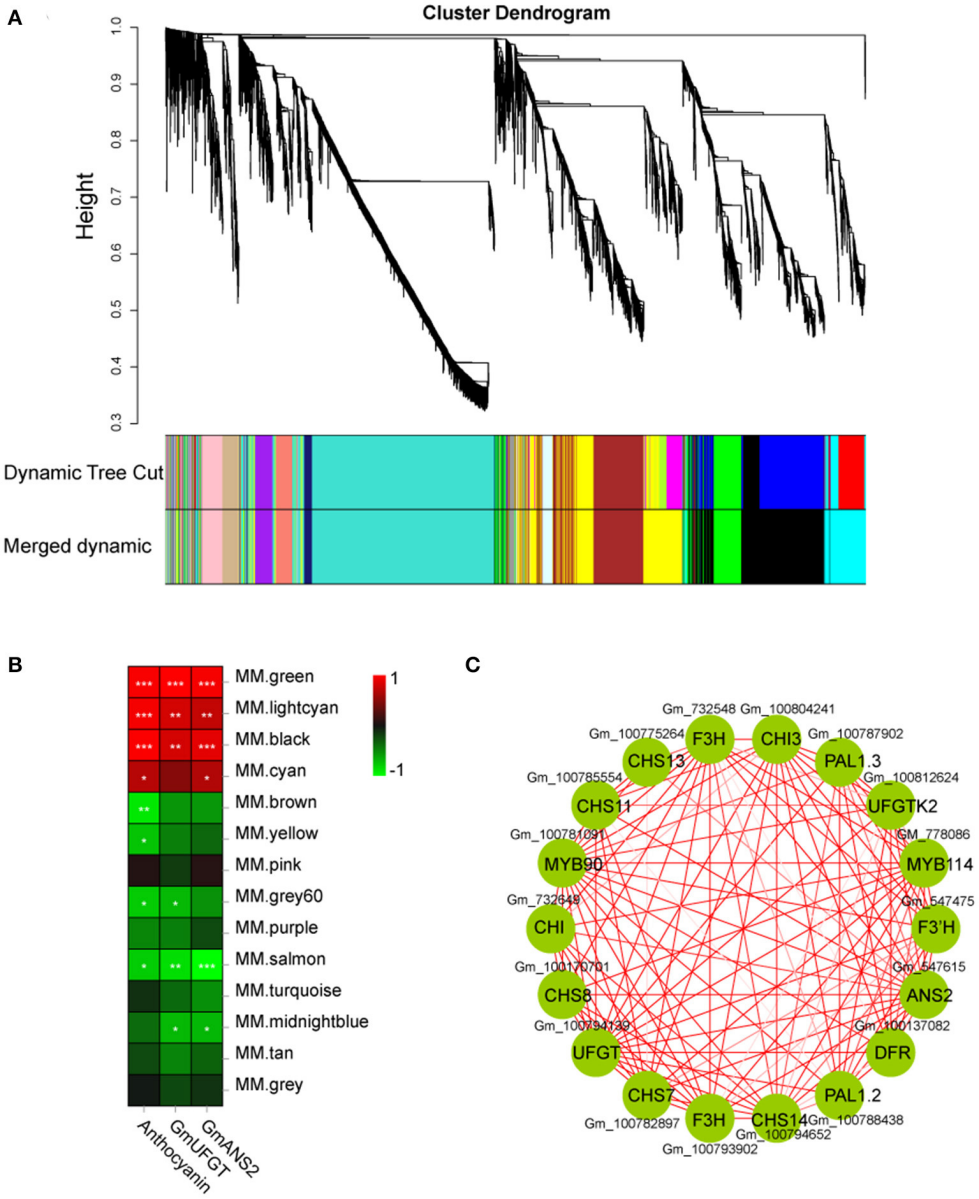
Weighted gene co-expression network analysis of DEGs identified in soybean sprout hypocotyl. **(A)** Hierarchical cluster tree showing 14 modules of co-expressed genes. Each of the DEGs is represented by a leaf in tree, and each of the modules by a major tree branch. The lower panel shows modules in designated colors. **(B)** Module-trait correlations and corresponding significant differences. The panel shows 14 modules. The color scale indicates module-trait correlations. Panel labeled “GmUFGT,” “GmANS2,” and “Anthocyanins” as a trait. **(C)** Cytoscape representation of co-expressed genes with the edge weights ≥.8 in the “MM.green” module. Member gene IDs are given.

DEGs were studied by performing weighted gene co-expression network analysis (WGCNA), identifying 14 modules (Figure 8A). The analysis of module-trait relationships showed that the “MM.green” module was highly correlated with the anthocyanin content in 24 samples (Figure 8B). Therefore, the genes related to this module might play a key role in the anthocyanin accumulation in soybean sprouts hypocotyl. Twenty-two genes that might be involved in anthocyanin biosynthesis were found in this module (“MM.green”), containing GmMYB90 (Gm_100781091) and GmMYB114 (Gm_778086) (Figure 8B). The “MM.green” module was further annotated by KEGG enrichment analysis. Diterpenoid biosynthesis, the pentose phosphate pathway, biosynthesis of secondary metabolites, and flavonoid biosynthesis were significantly enriched as a major pathway, and the metabolic pathways contained the largest number of genes (Supplementary Figure 4A). GO-based term classification was performed to provide insights into gene function. Thirty GO terms were found as enriched biological processes, the number of genes involved in metabolic process, antioxidant activity, and membrane-bounded organelle (Supplementary Figure 4B).

## Discussion

This study, from a multi-dimensional perspective, aimed to determine the molecular regulation mechanism of calcium on anthocyanin biosynthesis in soybean sprouts under blue light.

Calcium is a ubiquitous second messenger that is involved in secondary metabolism in plants. Spraying calcium is widely performed to improve the quality of crops in agricultural production (Chen et al., 2019). These results reflect those of Wang et al. (2016) who also reported that supplementing with Ca^2+^ can improve soybean sprout yield and increase bioactive substances. In this study, calcium notably increased the anthocyanin content in sprout hypocotyl at the initial germination stage, which was inhibited by LaCl_3_ and neomycin treatments (Figure 1), consistent with a previous study (Zhu et al., 2019). Interestingly, in addition to affecting the TAC, exogenous calcium also affected the composition of anthocyanins (Table 1). Previous studies have also shown that calcium affected the polyphenol profile and the metabolism of mevatin and quercetin in grape berries (Martins et al., 2020). This may be related to plant regulation metabolomic profile to respond to changes in the external environment. Besides, light-induced anthocyanin production is affected by changes in period, intensity, and wavelength. Blue light accelerating anthocyanin accumulation in plants has been widely reported (Tao et al., 2018; Zhang et al., 2018b; Zheng et al., 2019). In this study, blue light can more effectively stimulate the accumulation of anthocyanins in soybean hypocotyls (Figure 1). These studies prove that calcium is beneficial for the blue light-induced anthocyanin synthesis in “Dongnong 690” hypocotyl.

Furthermore, the relationship between the relative level and distribution of calcium and anthocyanin accumulation was explored. Exogenous calcium increased Ca^2+^ concentration in the root tip cells of barley sprouts, resulting in strong green fluorescence in cells, while LaCl_3_ and 2-aminoethoxydiphenyl borate inhibit calcium mobilization (Ma et al., 2019b), which is in accordance with the results in this study (Figure 2). Fluorescence images also show that anthocyanin accumulation areas near the vascular bundles have calcium enrichment in pear leaves (Zhai et al., 2019). The results shown in Figure 2 are consistent with the changing trend in anthocyanin content under corresponding conditions. Notably, a higher level of calcium is observed under blue light compared with that in the D and W treatments. This phenomenon was mainly attributed to phot2; and mutants and over-expressed transgenic lines related to phototrophic protein indicate that blue light specifically induces an increase in the cytoplasmic Ca^2+^ level in the hypocotyls of *A. thaliana* (Zhao et al., 2013). In addition, calcium presents temporal and spatial characteristics at different developmental stages and metabolic processes in plants. Yang et al. (2020) found that calcium showed particular distribution patterns at different stages in the anthers of *Impatiens balsamina*. In mung bean cotyledon cells, after calcium application, calcium precipitation increased in intercellular spaces and cell walls, and many spherical particles were observed, while LaCl_3_ inhibited Ca^2+^ influx into the cells (Zhou et al., 2018). Ma et al. (2019a) noted that calcium, in a specific distribution, participated in γ-aminobutyric acid signal transduction for phenolic compound accumulation in germinated hulless barley. Similar results were observed in this study. Calcium was mainly distributed in the cytoplasm and adjacent to the cell membrane, exogenous CaCl_2_, and its inhibitor affected the enrichment of calcium precipitation (Figure 3). This implies that calcium as an intracellular messenger depends on the kinetics of its spatia-temporal release from the calcium pool. However, the typical form of black precipitate in B is different from that in W and D (Figure 3), and may be related to a different light signal (Łabuz et al., 2016). Based on these studies, we speculate that calcium participates in the metabolism of anthocyanins in a specific spatiotemporal manner in “Dongnong 690” hypocotyl.

Calcium actively regulates anthocyanin biosynthesis through specific signaling pathways in most fruits and vegetables (Peng et al., 2016). The binding of IP3 to its receptors causes calcium channels on the organelles to open, thus releasing Ca^2+^ from the calcium store to the cytoplasm (Lovett et al., 2002). IP3 can induce isoflavone accumulation in soybean sprouts by upregulating the activity of isoflavone biosynthetic enzyme under UV-B radiation (Jiao et al., 2016). Similarly, there is a high correlation between IP3 content and anthocyanin accumulation in radish sprouts (Zhang et al., 2018a). In this study, exogenous calcium increased the IP3 content in hypocotyls, and there was a significant correlation with the anthocyanin content (Figure 4). Moreover, as the main calcium sensor, CaM plays an important role in decoding Ca^2+^ signals into downstream cellular physiological responses. In *Alternanthera bettzickiana*, the change in CaM activity is parallel to the increase in anthocyanin content at low temperatures; and chlorpromazine inhibits CaM activity and leads to a decrease in anthocyanins (Wang et al., 2005). Other researchers (He et al., 2020) have reported that calcineurin B-like proteins (CBLs) activate related kinases by sensing Ca^2+^, and then activate target genes related to anthocyanin biosynthesis. In this study, the changes in the CaM content were significantly consistent with the accumulation of anthocyanins under different conditions (Figure 4). These findings suggest that intracellular IP3-dependent Ca^2+^ and extracellular Ca^2+^ participate in blue light-induced anthocyanin synthesis in “Dongnong 690” hypocotyl, at least partially, through the Ca^2+^-CaM pathway. However, how CaM activates downstream target genes to promote the accumulation of anthocyanins requires more direct evidence.

In the calcium signal transduction pathway, transient Ca^2+^ oscillations in cells caused by external stimuli can be sensed by other Ca^2+^ sensors or binding proteins, such as calcium-dependent protein kinase (CDPK), calmodulin-like proteins (CMLs), and CBLs (Hashimoto and Kudla, 2011). Hierarchical cluster analysis showed that the expression pattern of calcium-responsive genes under blue light was different from that of other treatments (Supplementary Figure 5A). Among these DEGs, calmodulin and calcium-dependent protein kinase were significantly up-regulated, which is speculated to play an important role in the anthocyanin metabolism pathway. Calcium transport across organelle membranes and plasma membrane is tightly regulated by Ca^2+^-ATPase, calcium channel, V-type ATPase, and Ca^2+^/proton exchanger (De Freitas et al., 2012). As the main calcium transporter, upregulated calcium-transporting ATPase was involved in the regulation of calcium under blue light (Supplementary Figure 5A). How these Ca^2+^ sensors are involved in the regulation of blue light-induced anthocyanin biosynthesis and their interaction remains to be elucidated. Phytohormones that control anthocyanin accumulation have also been widely reported. Carvalho et al. (2010) proved that ABA played an active role in regulating anthocyanin biosynthesis in hormone mutants. Wang et al. (2019) reported that ethylene acts as a negative regulator in light-regulated anthocyanin biosynthesis in cabbage. Most upregulated ABA and downregulated ethylene related genes have been observed under blue light (Supplementary Figure 5B). Genes that encode auxin and salicylic acid-responsive elements have different expression modes and indicate that they have distinct functions in the regulation of anthocyanin biosynthesis through various transduction pathways. In general, some form of hormonal cross-talk may participate in pigment accumulation of soybean sprout hypocotyl.

Many studies have shown that calcium treatment can improve the expression of anthocyanin biosynthesis-related genes (Zhu et al., 2019; Yu et al., 2020). Xu et al. (2014) reported that calcium activated the key genes related to anthocyanin synthesis in the strawberry fruit, including FvDFR2, FvANS1, and FvUGT1. Contrariwise, EGTA and neomycin treatments markedly suppressed the activity, proteins, and gene expressions of GmCHS and GmIFS in soybean sprouts under UV-B irradiation (Jiao et al., 2016). Similarly, in this study, exogenous calcium promoted the expression of structural genes related to anthocyanin synthesis (Figure 5). These identified genes were also consistent with the WGCNA analysis results (Figure 8C). Besides, R2R3-MYB subgroup 6, namely, MYB75, MYB90, MYB113, and MYB114, contains factors that regulate anthocyanin biosynthesis in vegetative tissues (Gonzalez et al., 2008). The expressions of RsPAP1 and RsPAP2 in radish sprouts were stimulated by exogenous calcium, whereas EGTA and neomycin inhibited this process (Zhang et al., 2018a). In this study, the WGCNA analysis showed that GmMYB90 and GmMYB114 were also involved in the regulation of anthocyanin synthesis, and that they had high homology with AtPAP1 and AtPAP2 in *Arabidiposis thaliana* (Supplementary Figure 3). Furthermore, in the corresponding treatment, these MYBs and anthocyanin synthesis structural genes had similar expression patterns (Supplementary Figure 2). Although the specialized functions of these candidate genes have not been thoroughly characterized in this study, we provide valuable references for other researchers in the future. These studies support our findings that calcium regulates blue light-induced anthocyanin accumulation by upregulating the key genes involved in anthocyanin synthesis in “Dongnong 690” hypocotyl.

## Conclusion

Overall, exogenous calcium increased total anthocyanin content and affected the metabolism profile of the hypocotyls of soybean sprouts. The calcium signal, which is generated by the influx of extracellular calcium across the plasma membrane and the release of intracellular calcium pools, is involved in regulating blue light-induced anthocyanin biosynthesis in “Dongnong 690” hypocotyl. Simultaneously, calcium with certain temporal and spatial characteristics activates the expression of genes related to anthocyanin biosynthesis. This study may provide a better reference for production practices and help improve the quality of supplemental lighting used in artificial growth systems.

## Data Availability Statement

The datasets presented in this study can be found in online repositories. The names of the repository/repositories and accession number(s) can be found below: SRA, PRJNA719628.

## Author Contributions

JCu, NS, and GH designed the experiment. GH analyzed the data and wrote the first draft manuscript. XY, JS, GX, JCh, and HW accomplished the laboratory analysis and helped in data processing. All authors reviewed and approved the final manuscript.

## Conflict of Interest

The authors declare that the research was conducted in the absence of any commercial or financial relationships that could be construed as a potential conflict of interest.

## Supplementary Material

Supplementary Figure 1 Clusters of differentially expressed transcripts with expression profile changes. **(A,B)** Changes in gene expression profiles in 24 and 36 h treatment groups, respectively. The transcripts were divided into 20 clusters at each time point, representing distinct expression patterns. Colored profiles with significant differential expression at *p* < 0.05.

Supplementary Figure 2 Correlation analysis of the RNA-seq data and qRT-PCR. The RNA-seq and qRT-PCR results for these genes were highly correlated. The right y-axis shows the expression data of RNA-seq. The left y-axis indicates the corresponding gene relative expression levels detected by qRT-PCR. The x-axis shows different treatments at 24 or 36 h. D: dark. W: white light. B: blue light. La: blue light + 1 mM LaCl_3_.

Supplementary Figure 3 Phylogenetic tree comprising GmMYB90 and GmMYB114 in Glycine max and R2R3-MYB TFs in *A. thaliana*.

Supplementary Figure 4 KEGG pathway and GO annotation analyses of genes in MM. green module. **(A)** The X-axis is the gene ratio and the Y-axis is the pathway type. The bigger the bubble, the more different genes it contains. **(B)** The X coordinates in the diagram are GO entry names, and the Y coordinates are gene percent.

Supplementary Figure 5 Heat map representation of the expression patterns of genes related to calcium **(A)** and hormone **(B)** signal transduction. D: dark. W: white light. B: blue light. La: blue light + 1 mM LaCl_3_, 24 or 36 h.

Supplementary Table 1 List of primer sequences used in this study.

Supplementary Table 2 List of genes from different module.

Supplementary Table 3 Summary statistics of transcriptome sequencing.
