# Supplementary material for: Calcium Positively Mediates Blue Light-Induced Anthocyanin Accumulation in Hypocotyl of Soybean Sprouts
Source: Front Plant Sci. 2021 May 28;12:662091. doi: 10.3389/fpls.2021.662091 (PMC8194075; doi:10.3389/fpls.2021.662091)
Supplement: Supplementary Figure 1 — Clusters of differentially expressed transcripts with expression profile changes. (A,B) Changes in gene expression profiles in 24 and 36 h treatment groups, respectively. The transcripts were divided into 20 clusters at each time point, representing distinct expression patterns. Colored profiles with significant differential expression at p < 0.05. [file Data_Sheet_1.zip › Supplemtary Figure S1.PDF]

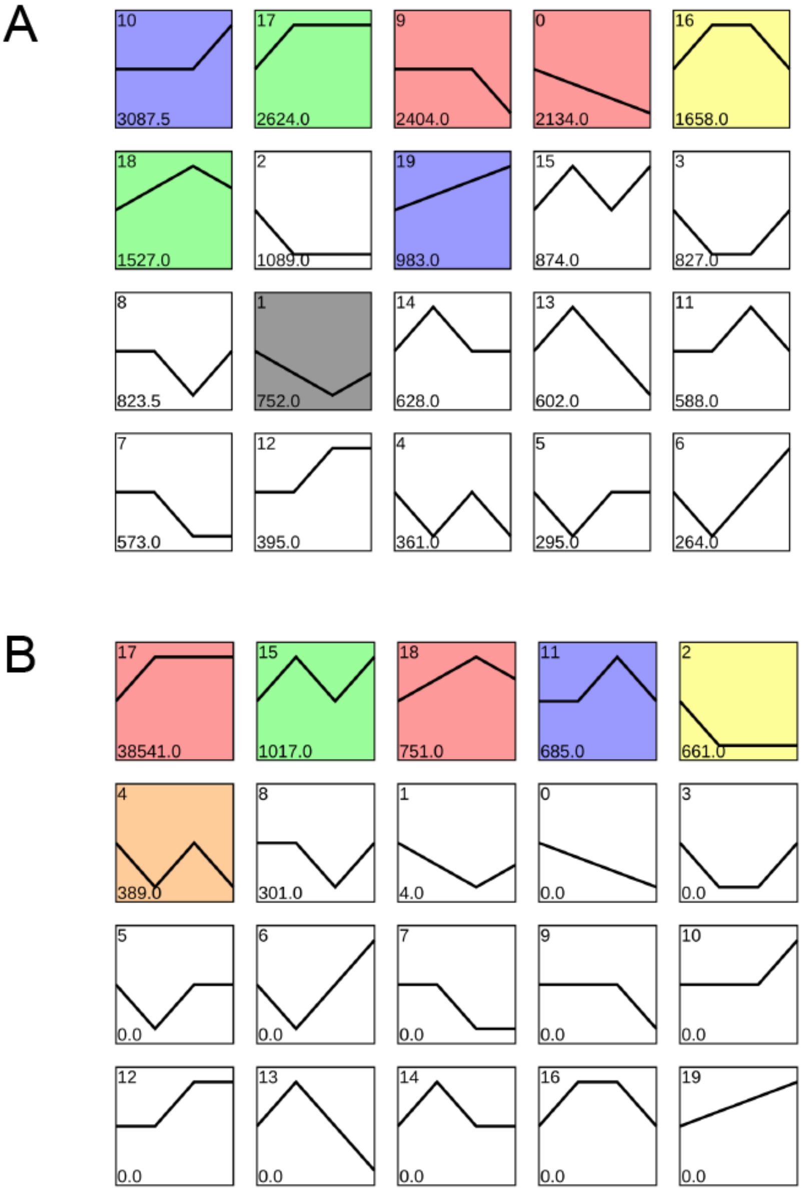

Supplementary Figure S1 Clusters of differentially expressed transcripts with expression profile changes. **(A)** and **(B)** Changes in gene expression profiles at 24 and 36h treatment groups, respectively. The transcripts were divided into 20 clusters at each time point, representing distinct expression patterns. Colored profiles with significant differential expression at p-value < 0.05.
