# Supplementary figures and images for: Calcium Positively Mediates Blue Light-Induced Anthocyanin Accumulation in Hypocotyl of Soybean Sprouts
Source: Front Plant Sci. 2021 May 28;12:662091. doi: 10.3389/fpls.2021.662091 (PMC8194075; doi:10.3389/fpls.2021.662091)

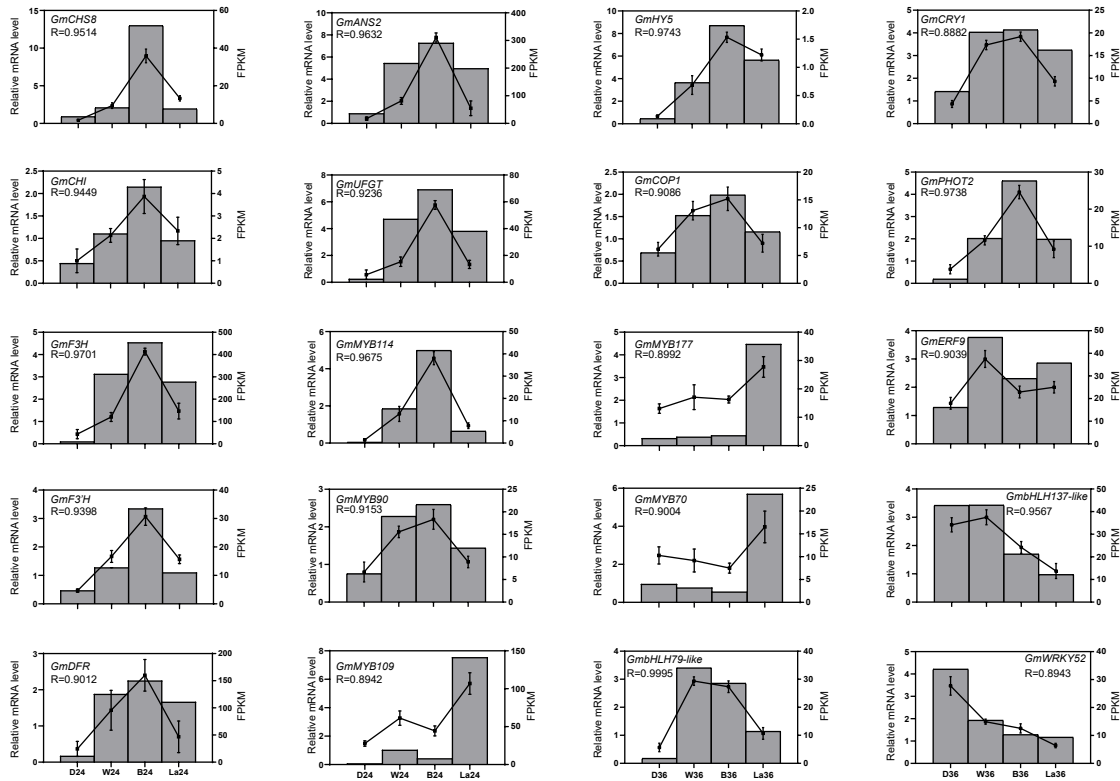

Supplement: Supplementary Figure 1 — Clusters of differentially expressed transcripts with expression profile changes. (A,B) Changes in gene expression profiles in 24 and 36 h treatment groups, respectively. The transcripts were divided into 20 clusters at each time point, representing distinct expression patterns. Colored profiles with significant differential expression at p < 0.05. [file Data_Sheet_1.zip › Supplemtary Figure S2.PDF]
